# A variational inequality of Kirchhoff-type in $\mathbb{R}^{N}$

**DOI:** 10.1186/s13660-018-1921-6

**Published:** 2018-11-28

**Authors:** Jiabin Zuo, Tianqing An, Wei Liu

**Affiliations:** 1 College of Science, Hohai University, Nanjing, P.R. China; 2 Faculty of Applied Sciences, Jilin Engineering Normal University, Changchun, P.R. China

**Keywords:** 35J87, 35J20, 49RJ40, 47G20, Mountain pass theorem, Kirchhoff-type, Variational inequality, Radial solutions

## Abstract

In this paper, we investigate the existence of nontrivial radial solutions for a kind of variational inequalities in $\mathbb{R}^{N}$. Our main technique is the non-smooth critical point theory, based on the Szulkin-type functionals.

## Introduction

Variational inequalities describe a lot of phenomena in the real world and have a wide range of applications in physics, mechanics, engineering etc.; see, for example, [1–3, 5–7, 9, 10, 12–14, 18]. This paper is concerned with a kind of variational inequalities in $\mathbb{R}^{N}$, the aim is to prove the existence of infinite radial solutions under suitable conditions.

Let $H^{1}_{O(N)}(\mathbb{R}^{N})$ be the Sobolev space of $O(N)$ invariant functions (see the definition in Sect. 3), and *B* be a closed convex set in $H^{1}_{O(N)}(\mathbb{R}^{N})$ with $0\in B$. Our problem, denoted by $(Q)$, is to find $u\in B$ such that

$$\begin{aligned}& \biggl(a + b \int_{\mathbb{R}^{N}}\bigl(\vert \nabla u \vert ^{2}+u^{2} \bigr)\,dx \biggr) \biggl( \int_{\mathbb{R}^{N}} \nabla u\cdot \nabla (v-u)\,dx \\& \quad {} + \int_{\mathbb{R}^{N}}u(v-u)\,dx \biggr) - \int_{\mathbb{R}^{N}}g(x,u) (v-u)\,dx \geq 0, \quad \mbox{for all } v\in B, \end{aligned}$$

where $a,b>0,N\geq 2$ and $g\in C(\mathbb{R}^{N}\times \mathbb{R}, \mathbb{R})$.

This problem is related to the obstacle problems, extensively studied due to the physical applications (see [15, 17]).

It is well known that the variational inequality is discussed in different ways in the case of regional bounded and unbounded. In [4], on the bounded interval $(0,1)$, a class of variational inequalities of Kirchhoff-type is discussed by applying the non-smooth critical point theory based on Szulkin functionals [16]. In [11], the authors study a kind of variational inequality defined on $(0,\infty )$. Motivated by the above work, in this paper we want to study the radial solutions of the problem $(Q)$ by using two kinds of theorem in [16]. Our research scope is an extension of some problems studied by [4] and [11]. Since the domain is unbounded and the continuous embedding $H^{1}( \mathbb{R}^{N})\rightarrow L^{p}(\mathbb{R}^{N})$ is not compact. We consider the symmetric method of the action of a group, similar to [8], to overcome this difficulty.

Meanwhile, suppose the function *g* satisfies:

$(g_{1})$
$\lim_{\vert u \vert \rightarrow 0}\frac{g(x,u)}{\vert u \vert }=0$ uniformly for $x\in \mathbb{R}^{N}$.
$(g_{2})$
For $1< p<2^{*}-1$ and there exists $c>0$ such that
  $$ \bigl\vert g(x,u) \bigr\vert \leq c\bigl(1+\vert u \vert ^{p} \bigr), \quad \mbox{for all } (x,u)\in \mathbb{R}^{N}\times \mathbb{R}, $$
  where
  $$ 2^{*}-1= \textstyle\begin{cases} \frac{N+2}{N-2}, &N\geq 3, \\ +\infty ,& N=1, 2. \end{cases} $$
$(g_{3})$
There is a constant $\mu >4$ such that
  $$ ug(x,u)\geq \mu G(x,u)= \int^{u}_{0}g(x,s)\,ds, \quad \mbox{for all } x\in \mathbb{R}^{N}, \mbox{ and } u\in {\mathbb{R}}^{N}. $$
$(g_{4})$
$\lim_{\vert u \vert \rightarrow +\infty }\frac{G(x,u)}{u^{4}} \rightarrow +\infty $ uniformly for all $x\in {\mathbb{R}}^{N}$.
$(g_{5})$
$g(x,u)=g(zx,u)$ for any $z\in O(N)$ and $(x,u)\in \mathbb{R}^{N}\times \mathbb{R}$.
$(g_{6})$
$g(x,u)=-g(x,-u)$ for any $(x,u)\in \mathbb{R}^{N}\times \mathbb{R}$.

We state the main result of this paper.

### Theorem 1.1

*If assumptions* $(g_{1})$*–*$(g_{5})$ *hold*, *then the problem* $(Q)$ *has a nontrivial radial solution in* *B*. *Furthermore*, *if the condition* $(g_{6})$ *holds*, *then the problem* $(Q)$ *has infinitely many pairs of nontrivial radial solutions in* *B*.

The structure of the paper is as follows. In Sect. 2, we review some preliminaries. Section 3 gives the proof of our main result.

## Szulkin-type functionals

Let X be a real Banach space and denote by $X^{*}$ its dual. Let $T=\varPhi +\psi $ with $\varPhi \in C^{1}(X,\mathbb{R})$ and let $\psi :X\rightarrow \mathbb{R}\cup \{+\infty \}$ be convex, lower semicontinuous. Then $T=\varPhi +\psi $ is a Szulkin-type functional. A point $u\in X$ is called critical if $\psi (u)\neq +\infty $ and

$$ \varPhi^{\prime}(u) (v-u)+\psi (v)-\psi (u)\geq 0 \quad \mbox{for all } v\in X, $$

or equivalently

$$ 0\in \varPhi^{\prime}(u)+\partial \psi (u)\quad \mbox{in }X^{*}, $$

where $\partial \psi (u)$ is called the subdifferential of *ψ* at *u*.

### Definition 2.1

([16])

The functional $T=\varPhi +\psi $ fulfills the $(PS)$ condition at level $c\in \mathbb{R}$; it can be written as $(PSZ)_{c}$ if every sequence $\{u_{n}\}\subset X$ such that $\lim_{n\rightarrow \infty }T(u_{n})=c$ and

$$ \bigl\langle \varPhi^{\prime}(u_{n}),(v-u_{n})\bigr\rangle _{X}+\psi (v)-\psi (u_{n}) \geq \varepsilon_{n} \Vert v-u_{n} \Vert \quad \mbox{for all } v\in X, $$

where $\varepsilon_{n}\rightarrow 0$, possesses a convergent subsequence.

### Lemma 2.2

([16], Mountain pass theorem)

*Suppose that* $T=\varPhi +\psi :X \rightarrow \mathbb{R}\cup \{+\infty \}$ *be a Szulkin*-*type functional and that*

(i) $T(0)=0$ *and there exist* $\alpha ,\rho >0$ *such that* $T(u)\geq \alpha $ *for all* $\Vert u \Vert =\rho $;
(ii) $T(e)\leq 0$ *for some* $e\in X$ *with* $\Vert e \Vert > \rho $.

*If* *T* *satisfies the* $(PSZ)_{c}$-*condition*, *then* *T* *has a critical value* $c\geq \alpha $ *which may be characterized by*

$$ c=\inf_{\gamma \in \varGamma }\sup_{t\in [0,1]}T\bigl(\gamma (t) \bigr), $$

*where* $\varGamma =\{\gamma \in c([0,1],X):\gamma (0)=0,\gamma (1)=e\}$.

### Lemma 2.3

([16], Corollary 4.8)

*Suppose that* $T=\varPhi +\psi :E\rightarrow \mathbb{R}\cup \{+\infty \}$ *is an even Szulkin*-*type functional and satisfies the* $(PSZ)_{c}$-*condition with* $T(0)=0$. *If* $E=X\oplus Y$, *where* *X* *is a finite dimensional*, *and assume also that*

$(A_{1})$
*there are constants* $\alpha ,\rho >0$ *such that* $T|_{\partial F_{\rho }\cap Y}\geq \alpha $;
$(A_{2})$
*for any positive integer* *k*, *there is* *k*-*dimensional subspace* $E_{k}\subset E$, *such that* $T(u)\rightarrow -\infty $ *as* $\Vert u \Vert \rightarrow +\infty$, $u\in E_{k}$.

*Then* *T* *has infinitely many pairs of nontrivial critical points*, *where* $F_{\rho }=\{u\in E:\Vert u \Vert <\rho \}$.

## The proof of the main result

Let

$$ H:=H^{1}\bigl(\mathbb{R}^{N}\bigr)=\bigl\{ u\in L^{2}\bigl(\mathbb{R}^{N}\bigr):\nabla u\in L ^{2} \bigl(\mathbb{R}^{N}\bigr)\bigr\} $$

be the Sobolev space with inner product and corresponding norm

$$ \langle u,v\rangle := \int_{\mathbb{R}^{N}}(\nabla u\nabla v+uv)\,dx, \qquad \Vert u \Vert = \biggl( \int_{\mathbb{R}^{N}}\bigl(\vert \nabla u \vert ^{2}+u^{2} \bigr)\,dx \biggr)^{\frac{1}{2}}. $$

Denote by $\Vert \cdot \Vert _{p}$ the norm of $L^{p}(\mathbb{R}^{N})$, i.e. $\Vert u \Vert _{p}= (\int_{\mathbb{R}^{N}}\vert u \vert ^{p}\,dx )^{\frac{1}{p}}$.

Let $O(N)$ is an orthogonal transformation group on $\mathbb{R}^{N}$. We have that

$$ E=H^{1}_{O(N)}\bigl(\mathbb{R}^{N}\bigr):=\bigl\{ u \in H\mid zu(x):=u\bigl(z^{-1}x\bigr)=u(x), \forall z\in O(N)\bigr\} $$

is a subspace of $H^{1}(\mathbb{R}^{N})$, and it is invariant. We note that the embedding $E\hookrightarrow L^{s}(\mathbb{R}^{N})$ is compact when $s\in (2,2^{*})$ by Corollary 1.26 of [19]. Define the functional $\varPhi :E\rightarrow \mathbb{R}$ by

3.1 $$ \varPhi (u)=\frac{1}{2}a\Vert u \Vert ^{2}+ \frac{1}{4}b\Vert u \Vert ^{4}-\varPsi (u), $$

where $\varPsi (u):=\int_{\mathbb{R}^{N}}G(x,u)\,dx$, and the indicator function of the set *B* as follows:

$$ \psi_{B}(u):= \textstyle\begin{cases} 0,& \mbox{if } u\in B, \\ +\infty ,& \mbox{otherwise}. \end{cases} $$

The function $\psi_{B}(u)$ is convex, proper, even, and lower semicontinuous. In order to show that $T=\varPhi +\psi_{B} $ is a Szulkin-type functional, we need the following proposition.

### Proposition 3.1

*Every critical point* $u\in E$ *of* $T=\varPhi +\psi_{B}$ *is a solution of* $(Q)$.

### Proof

Since $u\in E$ of $T=\varPhi +\psi_{B}$ is a critical point, we have

$$\begin{aligned} \varPhi^{\prime}(u) (v-u)+\psi_{B}(v)-\psi_{B}(u)\geq 0 \quad \mbox{for all } v\in E. \end{aligned}$$

It is clear that *u* belongs to *B*. If not, we get $\psi_{B}=+\infty $, and in the inequality above, setting $v=0\in B$ we get a contradiction. We fix $v\in B$. Since

$$\begin{aligned} \varPhi^{\prime}(u) (v-u)&=\bigl(a+b\Vert u \Vert ^{2}\bigr) \biggl( \int_{\mathbb{R}^{N}}\nabla u\nabla (v-u)\,dx + \int_{\mathbb{R}^{N}}u(v-u)\,dx\biggr) \\ &\quad {}- \int_{\mathbb{R}^{N}}g\bigl(x,u(x)\bigr) (v-u)\,dx\geq 0, \end{aligned}$$

*u* is a solution of $(Q)$. □

### Proposition 3.2

*Suppose that* *g* *satisfies the conditions* $(g_{1})$ *and* $(g_{2})$ *and* $\langle \varPsi^{\prime}(u),v\rangle =\int_{\mathbb{R}^{N}}g(x,u)v\,dx$, *then* $\varPhi \in C^{1}(E,\mathbb{R})$,

$$ \bigl\langle \varPhi^{\prime}(u),v\bigr\rangle = \biggl(a+b \int_{\mathbb{R}^{N}}\bigl(\vert \nabla u \vert ^{2}+u ^{2}\bigr)\,dx \biggr) \int_{\mathbb{R}^{N}}(\nabla u\nabla v+uv)\,dx -\bigl\langle \varPsi^{\prime}(u),v\bigr\rangle . $$

### Proof

By (3.1), we only need to prove that

$$ \varPsi \in C^{1}(H,\mathbb{R}),\quad \bigl\langle \varPsi^{\prime}(u),v\bigr\rangle = \int_{\mathbb{R}^{N}}g(x,u)v\,dx,\quad \forall u,v\in H. $$

Thus, we divide the whole proof into the following two steps.

*Step* 1. We verify that *Ψ* is a Gateaux derivative.

For small enough $\varepsilon >0$, using $(g_{1})$ and $(g_{2})$, there is a positive constant *c* depend on *ε* such that

3.2 $$ \bigl\vert g(x,u) \bigr\vert \leq \varepsilon \vert u \vert +c(\varepsilon )\vert u \vert ^{p} $$

for every $(x,u)\in \mathbb{R}^{N}\times \mathbb{R}$. For any $u(x),v(x)\in H$ and $0<\vert t \vert <1$, according to (3.2) and using the mean value theorem, there exists $\theta \in (0,1)$ such that

$$\begin{aligned} \frac{\vert G(x,u+tv)-G(x,u) \vert }{\vert t \vert } &=\bigl\vert g(x,u+\theta tv)v \bigr\vert \\ &\leq \varepsilon \vert u \vert \vert v \vert +\varepsilon \vert v \vert ^{2}+c(\varepsilon ) \bigl(\vert u+\theta tv \vert \bigr)^{p} \vert v\vert \\ &\leq \varepsilon \vert u\vert \vert v \vert +\varepsilon \vert v \vert ^{2}+2^{p}c(\varepsilon ) \bigl(\vert u \vert ^{p}\vert v \vert +\vert v \vert ^{p+1}\bigr). \end{aligned}$$

By the Hölder inequality, it follows that

$$\begin{aligned} h:=\varepsilon \vert u \vert \vert v \vert +\varepsilon \vert v \vert ^{2}+2^{p}c(\varepsilon ) \bigl(\vert u \vert ^{p}\vert v \vert +\vert v \vert ^{p+1}\bigr) \in L^{1}\bigl(R^{N}\bigr). \end{aligned}$$

So, by the Lebesgue dominated convergence theorem, we have

$$ \bigl\langle \varPsi^{\prime}(u),v\bigr\rangle = \int_{\mathbb{R}^{N}}g(x,u)v\,dx. $$

*Step* 2. We show that $\varPsi^{\prime}(\cdot ):H\rightarrow H^{*}$ is continuous.

Suppose that $u_{n}\rightarrow u$ in *H*. Since the imbedding $H\hookrightarrow L^{s}(\mathbb{R}^{N}) (2\leq s\leq 2^{*})$ is continuous, we see that, for each $s\in [2,2^{*}]$, there is a constant $\eta_{s}>0$ such that

$$ \Vert w \Vert _{s}\leq \eta_{s}\Vert w \Vert , \quad \forall w\in H^{1}\bigl(\mathbb{R}^{N}\bigr),\qquad u_{n}\rightarrow u \quad\mbox{in } L^{s}\bigl( \mathbb{R}^{N}\bigr). $$

Note that

$$\begin{aligned} \bigl\Vert \varPsi '(u_{n})-\varPsi '(u) \bigr\Vert &= \sup_{\Vert v \Vert \leq 1} \biggl\vert \int_{\mathbb{R}^{N}} \bigl(g(x,u_{n})-g(x,u) \bigr)v\,dx \biggr\vert \\ &\leq \sup_{\Vert v \Vert \leq 1} \int_{\mathbb{R}^{N}} \bigl\vert \bigl(g(x,u_{n})-g(x,u) \bigr) \bigr\vert \vert v\vert \,dx. \end{aligned}$$

According to the Hölder inequality, and Theorem A.4 in [19], we have

$$ \sup_{\Vert v \Vert \leq 1} \int_{\mathbb{R}^{N}} \bigl\vert \bigl(g(x,u_{n})-g(x,u) \bigr) \bigr\vert \vert v\vert \,dx\rightarrow 0 $$

as $n\rightarrow \infty $. So, we obtain $\Vert \varPsi '(u_{n})-\varPsi '(u) \Vert \rightarrow 0$, and thus the claim is proven. Consequently, $T=\varPhi +\psi_{B} $ is a Szulkin-type functional. □

It follows from $(g_{5})$ that *T* is $O(N)$-invariant, i.e. for all $(z,u)\in O(N)\times H$, $T(u)=T(zu)$, and the action of the group $O(N)$ on *H* is isometric, i.e. for all $(z,u)\in O(N)\times H$, $\Vert u \Vert =\Vert zu \Vert $. Furthermore, because of Lemma 2.2 and Theorem 1.28 of [19], we notice that *u* is a critical point of $T|_{E}$ if and only if *u* is a critical point of *T* in *H*. We will use the symmetric mountain pass theorem to obtain the critical points of the functional $T| _{E}$.

### Proposition 3.3

*If the continuous function f fulfills* $(g_{3})$ *and* $(g_{4})$, *then* $T=\varPhi +\psi_{B}$ *fulfills* $(PSZ)_{c}$-*condition for every* $c\in \mathbb{R}$.

### Proof

Fix $c\in \mathbb{R}$. Set $\{u_{n}\}\subset E$ such that

3.3 $$\begin{aligned}& T(u_{n})=\varPhi (u_{n})+\psi_{B}(u_{n}) \rightarrow c, \end{aligned}$$

3.4 $$\begin{aligned}& \varPhi^{\prime}(u_{n}) (v-u_{n})+ \psi_{B}(v)-\psi_{B}(u_{n})\geq -\varepsilon _{n}\Vert v-u_{n} \Vert ,\quad \forall v\in E, \end{aligned}$$

where $\varepsilon_{n}\rightarrow 0$ in $[0,\infty )$. According to (3.3), obviously, we notice that the sequence $\{u_{n}\}\subset B$. Setting $v=2u_{n}$ in (3.4) we have

$$ \varPhi^{\prime}(u_{n}) (u_{n})\geq - \varepsilon_{n}\Vert u_{n} \Vert . $$

Thus

3.5 $$ a\Vert u_{n} \Vert ^{2}+b\Vert u_{n} \Vert ^{4}- \int_{\mathbb{R}^{N}}g\bigl(x,u_{n}(x)\bigr)u_{n}(x) \,dx \geq -\varepsilon_{n}\Vert u_{n} \Vert . $$

On the basis of (3.3), for large enough $n\in N$, we get

3.6 $$ c+1\geq \frac{1}{2}a\Vert u_{n} \Vert ^{2}+\frac{1}{4}b\Vert u_{n} \Vert ^{4}- \int_{\mathbb{R}^{N}}G(x,u_{n})\,dx. $$

Multiply both sides of inequality (3.5) by $\mu^{-1}$, adding it to another inequality (3.6), and applying the condition $(g_{3})$. When $n\in N$ is sufficiently large, we have

$$\begin{aligned} &c+1+\frac{1}{\mu }\Vert u_{n} \Vert \\ & \quad \geq a \biggl(\frac{1}{2}-\frac{1}{\mu } \biggr)\Vert u_{n} \Vert ^{2}+b \biggl(\frac{1}{4}-\frac{1}{\mu } \biggr)\Vert u_{n} \Vert ^{4} \\ &\quad \quad {}- \int_{\mathbb{R}^{N}} \biggl(G\bigl(x,u_{n}(x)\bigr)- \frac{1}{\mu }g\bigl(x,u_{n}(x)\bigr)u _{n}(x) \biggr) \,dx \\ &\quad =a \biggl( \frac{1}{2}-\frac{1}{\mu } \biggr)\Vert u_{n} \Vert ^{2}+b \biggl( \frac{1}{4}-\frac{1}{\mu } \biggr) \Vert u_{n} \Vert ^{4} \\ &\quad \quad {}-\frac{1}{\mu } \int_{\mathbb{R}^{N}} \bigl(\mu G\bigl(x,u_{n}(x)\bigr)-g \bigl(x,u _{n}(x)\bigr)u_{n}(x) \bigr)\,dx \\ &\quad \geq a \biggl( \frac{1}{2}-\frac{1}{\mu } \biggr)\Vert u_{n} \Vert ^{2}+b \biggl( \frac{1}{4}- \frac{1}{\mu } \biggr)\Vert u_{n} \Vert ^{4}. \end{aligned}$$

Since $\mu >4$, the sequence $\{u_{n}\}$ is bounded in *B*. Then there exists a subsequence converging weakly in *E*. According to the compactness embedding $E\hookrightarrow \hookrightarrow L^{s}( \mathbb{R}^{N})$. Without loss of generality, assume

3.7 $$\begin{aligned}& u_{n}\rightharpoonup u \quad \mbox{in } E; \end{aligned}$$

3.8 $$\begin{aligned}& u_{n}\rightarrow u \quad \mbox{in } L^{s} \bigl(\mathbb{R}^{N}\bigr), s\in \bigl(2,2^{*}\bigr). \end{aligned}$$

By observing that *B* is weakly closed, we get $u\in B$. Let again $v=u$ in (3.4), we have

3.9 $$ \bigl(a+b\Vert u_{n} \Vert ^{2}\bigr) \langle u_{n},u-u_{n}\rangle_{E}+ \int_{\mathbb{R} ^{N}}g\bigl(x,u_{n}(x)\bigr) \bigl(u_{n}(x)-u(x)\bigr)\,dx\geq -\varepsilon_{n}\Vert u-u_{n} \Vert . $$

We use

3.10 $$ \bigl(a+b\Vert u_{n} \Vert ^{2}\bigr) \Vert u-u_{n} \Vert ^{2}=\bigl(a+b\Vert u_{n} \Vert ^{2}\bigr)\langle u-u_{n},u-u _{n}\rangle_{E}. $$

So, for large enough *n* and any $\varepsilon >0$, it follows from (3.9) and (3.10) that

$$\begin{aligned} &\bigl(a+ b\Vert u_{n} \Vert ^{2}\bigr)\Vert u-u_{n} \Vert ^{2} \\ &\quad \leq \bigl(a+b\Vert u_{n} \Vert ^{2}\bigr)\langle u,u-u_{n}\rangle_{E}+ \int_{\mathbb{R}^{N}}g(x,u_{n}) (u_{n}-u)\,dx+ \varepsilon_{n}\Vert u-u_{n} \Vert \\ &\quad \leq \bigl(a+b\Vert u_{n} \Vert ^{2}\bigr)\langle u,u-u_{n}\rangle_{E}+ \int_{\mathbb{R}^{N}}\bigl(\varepsilon \vert u_{n} \vert +c( \varepsilon )\vert u_{n} \vert ^{p}\bigr)\vert u-u_{n} \vert \,dx +\varepsilon_{n}\Vert u-u_{n} \Vert \\ &\quad \leq \bigl(a+b\Vert u_{n} \Vert ^{2}\bigr)\langle u,u-u_{n}\rangle_{E}+\varepsilon c _{1}+c( \varepsilon )\Vert u_{n}-u \Vert _{p+1}\Vert u_{n} \Vert _{p+1}^{p} +\varepsilon _{n}\Vert u-u_{n} \Vert \\ &\quad \leq \bigl(a+b\Vert u_{n} \Vert ^{2}\bigr)\langle u,u-u_{n}\rangle_{E}+\varepsilon c _{1}+c_{2}c( \varepsilon )\Vert u_{n}-u \Vert _{p+1} + \varepsilon_{n}\Vert u-u_{n} \Vert , \end{aligned}$$

where the constants $c_{1}$ and $c_{2}$ are independent of *n* and *ε*. By (3.7) and the fact that $\{u_{n}\}$ is bounded in *E*, we obtain

$$ \lim_{n}\bigl(a+b\Vert u_{n} \Vert ^{2}\bigr)\langle u,u-u_{n}\rangle_{E}=0. $$

Taking into account (3.8), $\Vert u_{n}-u \Vert _{p+1}\rightarrow 0$. Setting $\varepsilon_{n}\rightarrow 0^{+}$, then we have proved that

$$ \bigl(a+b\Vert u_{n} \Vert ^{2}\bigr)\Vert u-u_{n} \Vert ^{2}\rightarrow 0. $$

Consequently, we get ${u_{n}}\rightarrow u$ in *E*. This means that the proof of this conclusion has been completed. □

Now we give the proof of Theorem 1.1.

### Proof

By (3.2), for any $0<\varepsilon <\frac{a}{\eta_{2}^{2}}$ ($\eta_{2}\mbox{ is continuous imbedding constant } E\hookrightarrow L^{2}(\mathbb{R}^{N})$), we obtain

$$ \bigl\vert G(x,u) \bigr\vert \leq \int^{1}_{0}\bigl\vert g(x,tu)u \bigr\vert \,dt \leq \frac{\varepsilon }{2}\vert u \vert ^{2}+\frac{c( \varepsilon )}{p+1}\vert u \vert ^{p+1},\quad \mbox{for all }(x,u)\in \mathbb{R}^{N} \times \mathbb{R}. $$

The condition $(g_{4})$ implies $p>4$. Therefore, for small enough $\rho >0$, we have

$$\begin{aligned} T(u)&\geq \frac{1}{2}a\Vert u \Vert ^{2}+\frac{1}{4}b \Vert u \Vert ^{4}-\frac{\varepsilon }{2}\Vert u \Vert ^{2}_{2}-\frac{c(\varepsilon )}{p+1}\Vert u \Vert ^{p+1}_{p+1} \\ &\geq \frac{1}{2}\bigl(a-\eta_{2}^{2}\varepsilon \bigr)\Vert u \Vert ^{2}+\frac{1}{4}b \Vert u \Vert ^{4}-\frac{c(\varepsilon )}{p+1}c^{p+1}_{p+1}\Vert u \Vert ^{p+1} \\ &\geq \frac{1}{4}\bigl(a-\eta_{2}^{2}\varepsilon \bigr)\Vert u \Vert ^{2}+\frac{1}{4}b \Vert u \Vert ^{4}, \end{aligned}$$

for all $u\in \overline{F}_{\rho }$. Thus,

$$ T| _{\partial F_{\rho }}\geq \frac{1}{4}\bigl(a-\eta_{2}^{2} \varepsilon \bigr) \rho^{2}+\frac{1}{4}b\rho^{4}:=\alpha >0. $$

Let $\{e_{i}\}$ be a complete normal orthogonal basis of *E*. Take $X=\operatorname{span}\{e_{1},e_{2},\ldots,e_{n}\}$ and $Y=X^{\perp }$. Then $E=X\oplus Y$. Thus,

$$ T| _{\partial F_{\rho }\cap Y}\geq \alpha >0. $$

For every finite dimensional subspace $\widetilde{E}\subset E$, there exists $k\in N^{+}$ such that $\widetilde{E}\subset E_{k}$. Due to the equivalence of all norms in a finite dimensional space, for some positive constant $c_{4}$ we have

$$ \Vert u \Vert _{4}\geq c_{4}\Vert u \Vert , \quad \mbox{for all } u\in E_{k}. $$

According to the conditions $(g_{1})$, $(g_{2})$, and $(g_{4})$, we note that, for $D>\frac{b}{4c^{4}_{4}}$, there exists a positive constant $C(D)$ such that

$$ G(x,u)\geq D\vert u \vert ^{4}-C(D)\vert u \vert ^{2}, \quad \mbox{for all } (x,u)\in \mathbb{R}^{N} \times \mathbb{R}. $$

So, fixing $u_{0}\in B\backslash \{0\}\subset E_{k}$, and taking $u=su_{0}(s>0)$, we get

$$\begin{aligned} T(su_{0})&\leq\frac{1}{2}as^{2}\Vert u_{0} \Vert ^{2} +\frac{1}{4}bs^{4}\Vert u_{0}\Vert ^{4}-Ds^{4} \Vert u_{0} \Vert ^{4}_{4}+C(D)s^{2} \Vert u_{0} \Vert ^{2}_{2} \\ &\leq \frac{1}{2}as^{2}\Vert u_{0} \Vert ^{2}- \biggl(Dc^{4}_{4}s^{4}- \frac{1}{4}bs^{4} \biggr)\Vert u_{0} \Vert ^{4}+C(D)\eta^{2}_{2}s^{2}\Vert u_{0} \Vert ^{2}. \end{aligned}$$

Obviously, we have $T(su_{0})\rightarrow -\infty $ as $s\rightarrow + \infty $. Therefore, we take *s* ($e=su_{0}$) large enough such that $\Vert e \Vert >\rho $ and $T(e)<0$.

By Proposition 3.3, we know that *T* satisfies the $(PSZ)_{c}$-condition$(c \in \mathbb{R})$, and $T(0)=0$. So *T* has a critical value according to Lemma 2.2. We remark that the critical point $u_{1}\in E$ associated to the critical value *η* is nontrivial due to $T(u_{1})=\eta >0=T(0)$. From Proposition 3.1, we notice that $u_{1}\in B$ and it is a radial solution of $(Q)$.

If the condition $(g_{6})$ holds, then *T* is even. Similar to the previous discussion, we see that all conditions of Lemma 2.3 are satisfied. Therefore, the second conclusion of Theorem 1.1 is obtained. □

### Example 3.4

For $n=1,2,3,\ldots$ , considering $g(x,u)=u^{2n+1}\vert u \vert ^{\frac{2n+1}{2}}$, it is satisfied with all assumptions of Theorem 1.1.

## Conclusion

In this article, the existence of nontrivial radial solutions to problem $(Q)$ is established by using the variational methods under suitable conditions. We consider a variational inequality of Kirchhoff-type in $\mathbb{R}^{N}$, which improves the previous results. In order to overcome new difficulties, we need to adopt symmetric method of the action of a group in our paper.
